# Preimplantation genetic testing for four families with severe combined immunodeficiency: Three unaffected livebirths

**DOI:** 10.1186/s13023-024-03525-y

**Published:** 2025-01-09

**Authors:** Lingyun Zhang, Lei Feng, Hao Shi, Wenbin Niu, Yanchi Wang, Bei Bu, Yidong Liu, Xiao Bao, Wenyan Song, Haixia Jin, Yingpu Sun

**Affiliations:** 1 Center for Reproductive Medicine, The First Affiliated Hospital of Zhengzhou University, Zhengzhou, 450052 China; 2 Henan Key Laboratory of Reproduction and Genetics, The First Affiliated Hospital of Zhengzhou University, Zhengzhou, 450052 China; 3 Henan Provincial Obstetrical and Gynecological Diseases (Reproductive Medicine) Clinical Research Center, The First Affiliated Hospital of Zhengzhou University, Zhengzhou, 450052 China; 4 Henan Engineering Laboratory of Preimplantation Genetic Diagnosis and Screening, The First Affiliated Hospital of Zhengzhou University, Zhengzhou, 450052 China

**Keywords:** PGT-M, SCID, Karyomapping, Rare disease

## Abstract

**Purpose:**

Severe combined immunodeficiency (SCID) is a set of rare monogenic inherited diseases that together represent the most severe form of the primary immunodeficiency disease phenotype. Preimplantation genetic testing for monogenic defects (PGT-M) is an effective reproductive technology strategy to prevent disease-causing gene mutations from being transmitted to offspring. The aim of this study was to report the use of PGT-M strategy based on karyomapping in four families to avoid the birth of SCID children.

**Methods:**

Four couples underwent the PGT-M strategy due to SCID. The strategy of PGT-M started with a biopsy of the trophectoderm cells of embryos, and the whole genome was amplified by multiple replacement amplification (MDA). Then, the single nucleotide polymorphisms (SNPs) in the region upstream and downstream of the mutation site were subsequently identified via karyomapping, and the results were analyzed via SNPs linkage analysis. The aneuploids of the embryos were identified simultaneously. Finally, prenatal amniocentesis was used to verify the validity of the PGT-M results.

**Results:**

We identified three novel variants (case1: IL2RG c.720_726delGAGCCAC; case 3: RAG2 c.770 C > T; and case 4: LIG4 c.1347 A > T). All four couples with SCID pathogenic gene mutations were subjected to karyomapping linkage analysis, and embryos with the pathogenic gene mutation were successfully identified. Euploid blastocysts without pathogenic alleles were transplanted, and healthy offspring were ultimately born. Prenatal diagnosis also confirmed the validity of our results.

**Conclusion:**

This study revealed that karyomapping is an efficient approach for identifying SCID. Through PGT-M with karyomapping linkage analysis, healthy babies were born to families carrying mutations in the SCID pathogenic gene.

**Supplementary Information:**

The online version contains supplementary material available at 10.1186/s13023-024-03525-y.

## Introduction

Severe combined immunodeficiency (SCID) is a set of rare and life-threatening monogenic diseases that together represent the most severe form of the primary immunodeficiency disease phenotype. Patients with SCID frequently succumb to their condition within the first two years of their life unless the condition is recognized and treated by hematopoietic stem cell transplantation (HSCT), or gene therapy [1, 2]. To date, according to the 2022 updated phenotypical classification, more than 15 causative genes have been recognized as being associated with SCID [3, 4]. X-linked SCID (X-SCID) caused by interleukin 2 receptor gamma (IL2RG) gene mutations is the most common form of SCID [5]. Other genetic defects cause SCID that is mostly inherited in an autosomal recessive pattern, such as the Janus family tyrosine kinase 3 (JAK3) deficiency [6], interleukin 7 receptor alpha chain (IL-7Rα) deficiency [7], recombination activating gene 1 or 2 (RAG1/RAG2) deficiency [8], and DNA ligase IV (LIG4) deficiency [9].

Currently, the newborn screening and diagnosis of SCID in China are insufficient, which leads to infectious complications and a very poor prognosis in children; thus prevention of SCID is necessary to in reduce morbidity and mortality [10]. Couples with a history of SCID births are desperate to have healthy offspring. Hence, preventing the transmission of mutated SCID-associated genes to their offspring is of highly important and urgent. Preimplantation genetic testing (PGT) for monogenic defects (PGT-M) is an effective and revolutionary method of assisted reproductive technology in which single or several cells of in vitro fertilized embryos from patients with monogenetic defects are biopsied, genetic detection of the biopsied samples is performed, and unaffected embryos are selectively transferred to prevent disease inheritance [11, 12]. Furthermore, PGT-M technology can effectively prevent the long-term physical and psychological health concerns associated with induced abortion.

Owing to the small number of biopsy cells and low amount of DNA, the whole genome amplification (WGA) greatly involves a substantial risk of allele dropout (ADO), and can lead to severe misdiagnosis of compound heterozygous or autosomal dominant conditions [13–15]. Therefore, linkage analysis of genetic polymorphic markers is crucial for the diagnostic accuracy of PGT-M. Karyomapping provides an effective linkage analysis method for PGT-M by constructing haplotypes of family pathogenic genes via high SNP genotyping [16, 17]. To date, there are few reports of PGT-M for SCID [18, 19]. Reut et al. identified a new mutation DCLRE1C (p.C330*), that was responsible for causing SCID in a consanguineous family and demonstrated that PGT is feasible for patients with homozygosity alleles [18]. Jun et al. used PGT technology to prevent the inheritance of pathogenic variants of IL2RG (c.315T > A), and demonstrated that PGT is applicable in X-SCID [19]. However, there have been no reports on the use of PGT-M for compound heterozygous mutations in SCID.

In this study, we retrospectively analyzed the clinical data of four families that underwent PGT-M-assisted conception at the First Affiliated Hospital of Zhengzhou University, applied karyomapping technology to patients carring SCID gene mutations, and evaluated the accuracy and application value of the karyomapping linkage analysis strategy in PGT-M of SCID patients. In addition, a comprehensive analysis of the patients was performed and combined with the diagnostic results. Furthermore, we identified three novel variants (case 1: IL2RG c.720_726delGAGCCAC; case 3: RAG2 c.770 C > T; and case 4: LIG4 c.1347 A > T). Finally, three healthy live births were achieved in three families, validating the feasibility and effectiveness of our strategy. Overall, our work represents a crucial step towards the clinical application of karyomapping linkage analysis to prevent children being born with SCID and to have children free of SCID.

## Materials and methods

### Patients participating in PGT–M

We retrospectively collected the clinical data of four SCID families who underwent PGT-M-assisted conception at the Reproductive Medicine Center of the First Affiliated Hospital of Zhengzhou University from January 2016 to January 2022 due to SCID disease, including couples with SCID gene mutations and familial propositus, all of whom received genetic counselling and underwent assisted reproductive technology involving PGT-M. The gene detection results of revealed that four couples and the probands carried pathogenic mutations in the IL2RG, RAG2, and LIG4 genes, respectively. The characteristics of the four couples are listed in Table 1. All the data were acquired from the clinical reproductive medicine management system/electronic medical record cohort database (CCRM/EMRCD) at the Reproductive Medicine Center of The First Affiliated Hospital of Zhengzhou University in China. Follow up data were obtained from hospital records and telephone interviews with the patients or their relatives. This study was approved by the Ethics Committee of the First Affiliated University Hospital of Zhengzhou University (Approval number 2023-KY-0554-002).

**Table 1:** Characteristics of included SCID couples

Case ID	Female age	Female karyotype	Male age	Male karyotype	Mutation gene	Mutation site of female	Mutation site of male	ACMG	Reference source	No. of retrieved oocytes	No.of biopsied blastocysts
Case 1	28	46, XX	31	46, XY	IL2RG	c.720_726delGAGCCAC	—	LP	Couples’ son	13	7
Case 2	28	46, XX	32	46, XY	IL2RG	c.670 C> T	—	P	Female’s mother	13	5
Case 3	35	46, XX	34	46, XY	RAG2	c.770 C> T	—	VUS	Female’s mother	13	5
						—	c.1396 C> T	LP	Male’s mother		
Case 4	36	46, XX	37	46, XY	LIG4	c.1347 A> T	c.1347 A> T	VUS	Couples’ fetus	9	5

Abbreviation: SCID, Severe combined immunodeficiency; ACMG, American College of Medical Genetics and Genomics; LP, likely pathogenic; P, pathogenic; VUS, uncertain significance

### Gene mutation detection

Genomic DNA was extracted from the peripheral blood samples of the couples and the probands with a QIAamp DNA Blood Mini Kit (Qiagen, Germany), and the extracted genomic DNA was sequenced via whole exome sequencing (WES), including library construction, probe capture, and next-generation sequencing (NGS). The SCID gene mutations detected by WES were verified by Sanger sequencing. The pathogenicity of the mutation was evaluated according to the American College of Medical Genetics and Genomics (ACMG) genetic variation classification criteria [20].

### Gene mutation studies

Variant Interpretation was performed with Mutation Taster (http://www.mutationtaster.org/), PolyPhen-2 (http://genetics.bwh.harvard.edu/pph2/), SIFT (http://sift.jcvi.org), and the Rare Exome Variant Ensemble Learner (REVEL) were used to predict the pathogenicity of the mutation sites. The gnomAD exomes database (https://gnomad.broadinstitute.org/) was used to calculate the frequency of corresponding variants in the total population. The pathogenicity interpretation of the variants followed ACMG recommendations [20].

### Study Workflow

**Fig. 1:**
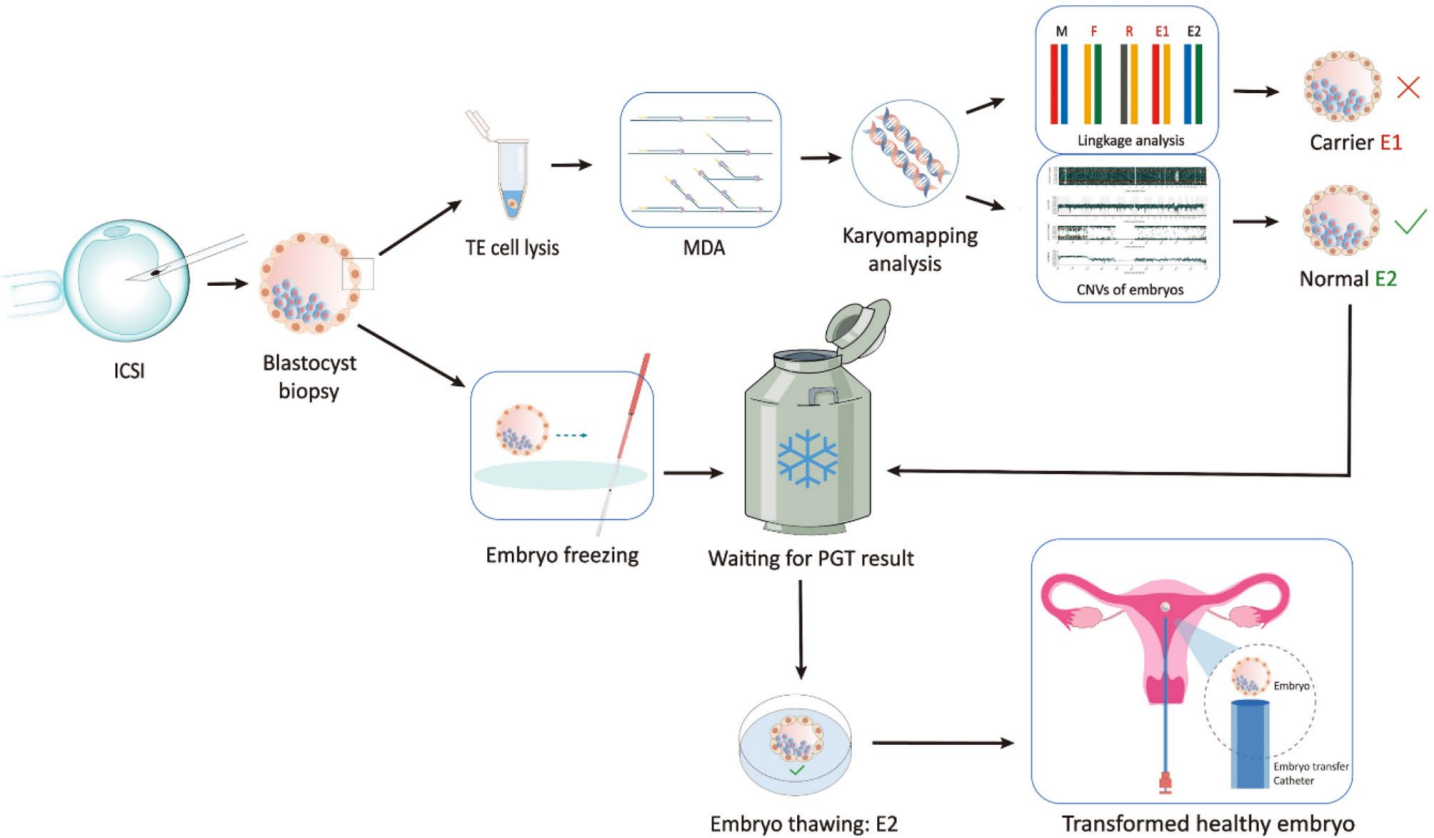
Workflow of PGT for severe combined immunodeficiency disease by karyomapping. ICSI, intracytoplasmic sperm injection; TE, trophoblastic ectodermal; MDA, multiple replacement amplification; PGT, preimplantation genetic testing for monogenic defects; E, embryo; CNV, copy number variation

The workflow of this study strategy is shown in Fig. 1. First, the embryonic trophoblastic ectodermal (TE) cells were lysed with lysis buffer, and the DNA of the TE cells was extracted for whole genome amplification (WGA) via multiple displacement amplification (MDA) [21, 22]. Second, the amplified DNA was applied to a karyomap SNP chip for the following analysis. The SNPs in the regions upstream and downstream of the mutation site (approximately 2.0 Mb) were subsequently designed for haplotype analysis to determine the carrier status of the biopsied embryo. Our screening conditions for information SNPs that can be used for linkage analysis are as follows: (i) The proband is the homozygous genotype (AA or BB) for the allele at this locus. (ii) In SCID couples, one person was heterozygous (AB), and the other was homozygous genotype for the allele. Simultaneously, the TE cells of the biopsied embryos were detected via copy number variation (CNV) to screen aneuploidy. Finally, according to the haplotype comparison between the embryo and the parents and proband, healthy euploid embryos without pathogenic mutations were successfully identified and transplanted into the mother’s uterus after resuscitation.

### Ovulation induction

According to the standard scheme, controlled superovulation was carried out at the Reproductive Medicine Center of the First Affiliated Hospital of Zhengzhou University. After human chorionic gonadotropin injection, the eggs were collected and fertilized via intracytoplasmic sperm injection (ICSI) and cultured the embryos according to the standard protocol.

### Blastocyst biopsy and cryopreservation

When the embryo developed to the blastocyst stage (5 ~ 6 days), 3 ~ 5 TE cells were obtained from the hatched blastocyst trophoblast cells via a biopsy needle. After the blastocyst biopsy, the blastocyst was cryopreserved via vitrification cryopreservation. The biopsy cells were subjected to whole-genome amplification.

### Single–cell whole genome amplification (WGA) and karyomap gene chip detection

After whole genome amplification of TE cells via multiple displacement amplification (MDA), haplotyping was performed. The technical process of this step followed the standard procedure provided by the QIANGEN REPLI-g Single Cell Kit (Qiagen, Germany). Then, the DNA extracted from peripheral blood and the WGA products of blastocyst biopsy were analyzed via the HumanKaryomap-12 Bead Chip microarray (Illumina, USA) according to the manufacturer’s instructions. Finally, a HiScan AQ chip scanner (Illumina, USA) was used to scan the original data. The karyomap chip data were analyzed with BlueFuse Multi Software (Illumina, USA), and haplotype linkage analysis, blastocyst chromosome aneuploidy analysis and blastocyst recombinant genotype analysis were carried out.

### Linkage analysis and CNVs detection with karyomapping

We detected the genomes of couples and their references in four SCID mutation-carrying families, identified alleles linked to pathogenic genes, and successfully established disease-associated haplotypes. According to the genome sequence of the biopsied blastocyst TE cells, the heterozygous or homozygous SNP readouts in the 2 Mb region adjacent to the mutant gene were used to identify the disease-carrier allele in the blastocyst (Table S1). Moreover, BlueFuse Multi software was used to identify the whole genome CNVs of blastocysts with the raw information SNP data from the HumanKaryomap-12 Bead Chips.

### Frozen embryo transfer (FET) and prenatal diagnosis of amniocentesis

After the mutant gene carrier status of the blastocysts was detected via karyomapping, healthy blastocysts without mutations were selected for resuscitation and transplantation. After 14 days and 18 days, the peripheral blood of the female patients was taken to measure the HCG value and confirm a biochemical pregnancy, and 35 days after transplantation, the fetal heart was detected by ultrasound to confirm the clinical pregnancy. Amniocentesis was performed at approximately 18 weeks of pregnancy, and the genes of amniotic fluid cells were detected.

All procedures were subject to repeated verification by at least two experimental technicians. Figure 1 shows the operation flow of the karyomapping linkage analysis of the mutant genes.

## Results

### Characteristics of the included couples

**Fig. 2:**
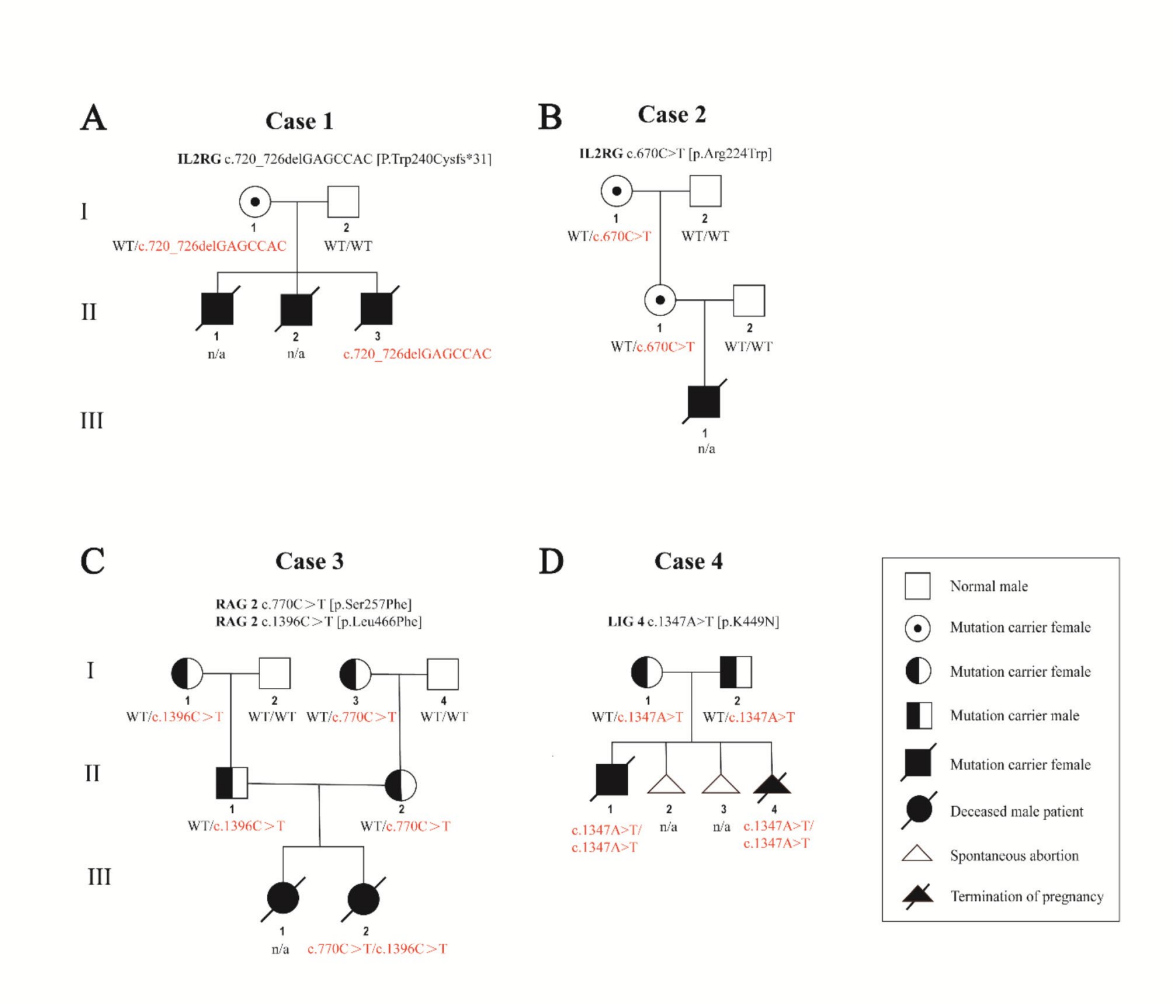
Pedigree of the four families carring SCID pathogenic genes. The upper right box shows the symbols and their meanings. The genotypes are displayed below the family members. WT, wild type. n/a, not available

A total of four families were included in our retrospective analysis. All four families were involved in three SCID pathogenic genes respectively. Twenty-two embryos from four PGT-M cycles were biopsied from the four couples, and three healthy live births occurred in three families. We identified three novel variants in the IL2RG, RAG2, and LIG4 pathogenic genes, and the gene information is shown in Table 2. Pedigrees of the four families are shown in Fig. 2.

**Table 2:** Overview of the related variant in the 4 families

Case ID	Mutation gene	Gene Position	Exon	cDNA change	Protein change	Variant type	Known/novel variants	Inheritance	MutationTaster^a^	PolyPhen-2^b^	SIFT^c^	REVEL^d^	gnomAD Exomes^e^
1	IL2RG	X:70,329,115	5	c.720_726delGAGCCAC	p.Trp240Cys fs*31	Frameshift	Novel	XR	D	N/A	N/A	N/A	N/A
2	IL2RG	X:70,329,165	5	c.670 C> T	p.Arg224Trp	Missense	rs869320658	XR	D	PD	D	D	N/A
3	RAG2	11:336,149,149	2	c.770 C> T	p.Ser257Phe	Missense	Novel	AR	D	PD	D	D	N/A
3	RAG2	11: 36,614,323	2	c.1396 C> T	p.Leu466Phe	Missense	rs1590713653	AR	D	PD	D	D	N/A
4	LIG4	13:108,862,270	2	c.1347 A> T	p.Lys449Asn	Missense	Novel	AR	D	PD	D	D	N/A

Abbreviation: XR, X-linked recessive; AR, autosome recessive; D, deleterious; PD, predicted deleterious; N/A, not available; PolyPhen-2, polymorphism phenotyping v2; SIFT, sorting intolerant from tolerant; REVEL, rare exome variant ensemble learner; gnomAD, genome aggregation database

^a^ Variant effect predicted by Mutation Taster

^b^ Variant effect predicted by PolyPhen-2

^c^ Variant effect predicted by SIFT

^d^ Variant pathogenicity predicted by REVEL

^e^ Frequency of corresponding variants in the total population of gnomAD Exomes

- **Case 1** The couple had three male infants, all of whom presented with recurrent infections with severe clinical signs of immunodeficiency and survived only 4–9 months. Whole exome sequencing revealed that the proband had a hemizygous variant in the IL2RG gene (c.720_726delGAGCCAC, p.Trp240Cys fs*3). The female was a carrier of the same variant, and the male partner did not carry this variant (Fig. 2A). This variant was classified as a likely pathogenic variant according to ACMG guidelines [20] (Table 1). The variant in exon 5 of the IL2RG gene and the mutation is novel, and prediction by Mutation Taster suggested that the variant was deleterious (Table 2).
- **Case 2** The couple had a child with immunodeficiency who died in the fourth month of life. Whole exome sequencing results revealed that the female partner was a carrier of a variant in the IL2RG gene (c.670 C > T, p.Arg224Trp), which originated from her mother (Fig. 2B). The variant was classified as a pathogenic variant according to ACMG guidelines, and in silico prediction by Mutation Taster, PolyPhen-2, SIFT, and REVEL suggested that the variant was deleterious.
- **Case 3** The couple had given birth to two consecutive children suspected of immunodeficiency. The eldest daughter died of cough, fever, diarrhea, and other causes at the age of 14 months, immunodeficiency was suspected, but the daughter was not genetically tested. Eight months after birth, the youngest daughter developed immune deficiency symptoms such as cough, diarrhea, fever, otitis media, low growth and development, and a low T cell ratio, and died at the age of 6. We obtained the whole exome sequencing results of only the youngest daughter, whose test at another hospital revealed compound heterozygous RAG2 mutations (c.770 C > T, p.Ser257Phe and c.1396 C > T, p.Leu466Phe). Given the lack of access to the DNA of the two daughters, whole exome sequencing was performed on the couple and revealed that the female partner was heterozygous for the RAG2 c.770 C > T, p.Ser257Phe mutation (inherited from her mother), and the male partner was heterozygous for the RAG2 c.1396 C > T, p.Leu466Phe mutation (inherited from his mother) (Fig. 2C). The c.770 C > T mutation in the female partner is a novel variant in exon 2 of RAG2 and is classified as an uncertain significance according to the ACMG guidelines. The c.1396 C > T mutation in the male partner is a known variant in is a novel variant in the exon 2 of the RAG2 and is classified as a likely pathogenic variant according to the ACMG guidelines. The results of Mutation Taster, PolyPhen-2, SIFT, and REVEL prediction suggested that the two mutations were deleterious (Table 2).
- **Case 4** The couple had given birth to a child suspected of immunodeficiency. Whole exome sequencing results revealed that the child had a homozygous mutation in the LIG4 gene (c.1347 A> T, p.K449N). Subsequently, we detected that the couple were heterozygous carriers of the same mutation (LIG4 c.1347 A> T, p.K449N) (Fig. 2D). When the couple get pregnant for the fourth time, they opted for pregnancy termination for the fetus with homozygous mutation in the LIG4 gene. The mutation is a novel variant and classified as an uncertain significance variant according to the ACMG guidelines; the Mutation Taster, PolyPhen-2, SIFT, and REVEL predictions suggested that the mutation was deleterious (Table 2).

All mutations were confirmed by Sanger sequencing.

### Determination of informative SNPs

Before haploid analysis, we determined the chromosome locations of the pathogenic genes (IL2RG, RAG2, LIG4) in all four families and selected a region approximately 2.0 Mb in size covering the upstream and downstream regions surrounding the gene location as the linkage analysis region (Supplementary Table S1). For example, in case 2, the mutation gene IL2RG is located on the Xq13.1 segment; the start and stop positions are 71,107,404 and 71,111,577, respectively, and the length of the region is approximately 4.2 kb. Therefore, we set the analysis zone for IL2RG as the region from 70,107,404 to 72,111,577, and the size was approximately 2.0 Mb, approximately. The SNP information for each family is shown in Supplementary Tables S2-6.

### Haplotype analysis via karyomap microarray

Haplotypes related to the selected mutant alleles were identified from the genotypes of parents and probands. Our analysis showed that haplotypes related to each mutant allele were found in all four families. The number of informational SNPs used to establish haplotypes in families1-4 was 14, 8, 21, and 30, respectively.

**Fig. 3:**
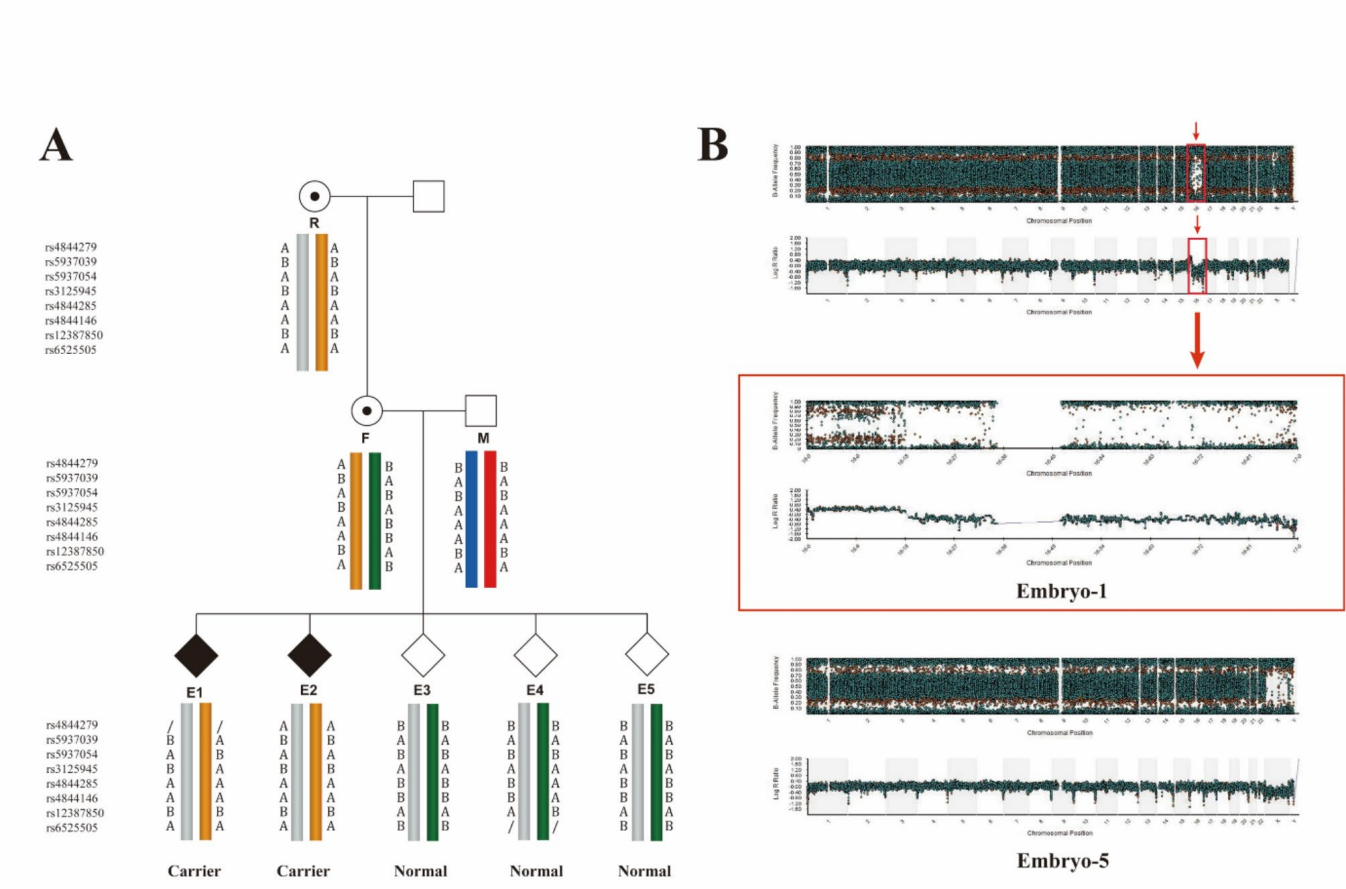
PGT-M strategy for severe combined immunodeficiency disease. (**A**) Pedigree diagram and linkage analysis identified for the disease-carring allele. We sequenced the amplified genomes from each embryo, couple, and reference. This is the pedigree diagram of case 2 in the PGT-M cycle. The SNPs in the upstream and downstream regions flanking the IL2RG gene within a range of 2 Mb are shown on the left. The heterozygous SNPs (AB) for the female and homozygous SNPs (AA or BB) for the male and the female’s mother were applied in the linkage analyses. The yellow bars indicate the pathogenic allele in the female’s mother passed by the female to embryos E1 and E2. (**B**) Results of CNV detection in abnormal embryos. Top: CNVs of abnormal embryos E1 by karyomapping microarray; Middle: CNVs on the chromosome 16 of abnormal E1 embryos. Bottom: CNVs of normal E5 embryos identified via a karyomapping microarray. M, male; F, female; R, reference, female’s mother; E, embryo; SNP, single nucleotide polymorphism; CNV, copy number variation

Next, we describe the derivation strategy of linkage analysis using case 2 as an example. As shown in Fig. 3A, there were a total of 8 available informational SNPs. We collected and fertilized 13 metaphase II oocytes and biopsied the TE cells of the blastocysts. Our genetic diagnosis showed that the female had a heterozygous variation in the IL2RG gene (IL2RGc.670 C > T, p. Arg224Trp), which was inherited from her mother. Informational SNPs are the main markers used for judging the chromosomes inherited by embryos from their parents. In case 2, our screening criteria for informational SNPs that could be used for linkage analysis were as follows: (i) The reference is homozygous (A/A or B/B) for the allele at the locus. (ii) The female partner is heterozygous (A/B) and the male partner is homozygous (A/A or B/B) for the allele. Therefore, we chose the SNP carried by the female partner (Fig. 3A, Female) as the representative SNP of the A/B genotype and those carried by her husband (Fig. 3A, Male) and her mother (Fig. 3A, Reference) as the representative SNPs of the A/A or B/B genotypes. For case 2, as shown in Fig. 3A, the female was A/B at the locus rs4844285, the reference was A/A, and the male was A/A. Thus, it could be inferred that the A allele of the female inherited from her mother was pathogenic, and the other allele B of the female was a nonpathogenic. The homozygous A/A male could only pass the A allele to the embryo, therefore, in the biopsy embryos E1 and E2 with homozygous allele A/A, one A was a non-pathogenic allele from the male, and the other A was pathogenic allele from the female. Therefore, the embryo E1 and E2 were carriers of the IL2RG mutation and were not transplanted. Similarly, embryonic E4 (A/B) has a nonpathogenic allele B from the female and a nonpathogenic allele A from the male. Embryo E3 and E5 (B/B) only nonpathogenic allele B were inherited from the female. Therefore, among the five embryos, E1 and E2 were IL2RG mutation carriers, and E3-E5 were nonmaternal mutation carriers. The aneuploids of the embryos were identified simultaneously, 4 embryos were normal, embryo E1 had abnormal chromosomes (Fig. 3B; Table 3).

**Table 3:** The results of haplotype analysis and CNVs of embryos

Patient ID	Genetic type	Cycle	Embryo	CNV	Mutation carrier status	Information SNP(2 M)	Recommendation	Clinical outcome
Case 1	XR	1	1	46,XN, del(4)(q28.3-qter)	Carrier (maternal)	14	Not Transferred	Abandoned
		1	2	46,XN	Carrier (maternal)	14	Not Transferred	Abandoned
		1	3	46,XN,5q-	Carrier (maternal)	14	Not Transferred	Abandoned
		1	4	46,XN	Normal	14	Transferred	Live birth
		1	5	46,XN	Carrier (maternal)	14	Transferred	Cryopreservation
		1	6	45,XN,-16	Carrier (maternal)	13	Not Transferred	Abandoned
		1	7	46,XN	Carrier (maternal)	14	Not Transferred	Abandoned
Case 2	XR	1	1	46,XN, dup(16)(p13.3-p12.3),del(16)(p12.3-qter)	Carrier (maternal)	7	Not Transferred	Abandoned
		1	2	46,XN	Carrier (maternal)	8	Not Transferred	Abandoned
		1	3	46,XN	Normal	8	Transferred	Live birth
		1	4	46,XN	Normal	7	Transferred	Cryopreservation
		1	5	46,XN	Normal	8	Transferred	Cryopreservation
Case 3	AR	1	1	46,XN	Carrier (all)	21	Not Transferred	Abandoned
		1	2	44,XN,-15,-20	Carrier (paternal)	21	Not Transferred	Abandoned
		1	3	45,XN,-5	Carrier (all)	21	Not Transferred	Abandoned
		1	4	46,XN	Carrier (maternal)	16	Transferred	No pregnancy
		1	5	46,XN	Carrier (paternal)	19	Transferred	Live birth
Case 4	AR	1	1	47,XN,+1	Carrier (all)	27	Not Transferred	Abandoned
		1	2	45,XN,-14	Carrier (all)	28	Not Transferred	Abandoned
		1	3	45,XN,-11	Carrier (maternal)	28	Not Transferred	Abandoned
		1	4	45,XN,-13	Carrier (paternal)	25	Not Transferred	Abandoned
		1	5	45,XN,-7	Carrier (paternal)	19	Not Transferred	Abandoned

Abbreviation: CNV, copy number variation; SNP, single nucleotide polymorphism; XR, X-linked recessive; AR, autosomal recessive

A detailed analysis of the pathogenic gene carrier status of embryos in four families is shown in the Supplementary Tables S2-6.

### Embryo selection of healthy embryos and results of frozen embryos transfer

Based on the aneuploidy and linkage analysis of embryos in each family, transferable and nontransferable embryos were identified. Unfortunately, all the biopsied embryos in the case 4 carried pathogenic genes and there were no transferable embryos. Cases 1, 2, and 3 all presented normal transferable embryos, among which we selectively transferred euploid embryos with higher morphological grades, and the results are shown in Table 3. Subsequently, amniotic fluid samples were collected from women with definite clinical pregnancies for fetal karyotype analysis, and the results of PGT-M were confirmed by detecting gene mutations in gDNA in amniotic cells.

Finally, cases 1 and 2 each gave birth to one healthy baby that did not carry gene mutations according to amniotic fluid screening. The first transplantation in case 3 failed, and a heterozygous baby with a normal phenotype but carrying the male RAG2 gene mutation was born after the second transplantation.

## Discussion

In this study, we reported an efficient and feasible PGT-M strategy based on karyomapping linkage analysis to identify the carrier status of pathogenic gene mutations in embryos and confirmed the effectiveness of this strategy through a retrospective study of included families. There are few reports on PGT-M for monogenic SCID in the literature [18, 19]. In our study, the mutations detected in the four SCID families had X-linked and autosomal recessive inheritance modes. Moreover, we reported five variants in three pathogenic genes (IL2RG, RAG2, and LIG4), including two known variants and three novel variants. Preimplantation diagnoses were made via karyomapping in all four SCID families. Through linkage analysis and aneuploidy tests after TE cell biopsy, and after comprehensive consideration of embryo growth rate, embryo morphology and PGT results, we successfully selected transferable embryos from various families and implanted the embryos into the mother through FET thawing. Ultimately, three healthy babies were born successfully.

As a monogenic genetic disease, SCID is mainly inherited through X-linked recessive inheritance or autosomal recessive inheritance, and both males and females in the SCID family can carry pathogenic genes, posing a severe threat to future generations. Therefore, preventing the vertical transmission of pathogenic mutations in family genes to offspring is vital. Currently, HSCT is commonly used to treat postnatal SCID, and studies have shown that gene therapy is also successfully used in SCID patients [23, 24]. However, as the success rate of transplantation is affected by the age of patients and the degree of infection before transplantation, these two techniques still have limitations [2, 25, 26]. The age of the child and the status of the infection before transplantation are important factors affecting the success of HSCT, so the survival rate can only be improved if the transplant is performed as soon as possible before the child is seriously infected, but many children are not so lucky [25]. In addition, the transplantation of hematopoietic stem cells is limited by the source of the donor, the health status of the child and other factors, and cannot completely address the problem of immune reconstitution. The drawbacks of the other treatment method, gene therapy, are also obvious, while improving the immune function of children, it also increases the risk of leukemia and lymphoma [2]. Currently, PGT-M is an effective technique for preventing the vertical transmission of pathogenic genes to offspring. In this situation, the karyomapping technique is an ideal strategy for analyzing a small number of cells from biopsied embryos for PGT-M. This technology uses the karyomap chip covering approximately 300,000 SNP sites in the genome to construct haplotypes of pathogenic genes in families and applies the strategy of linkage analysis to PGT detection of single-gene diseases [27]. Because the karyomap chip covers the entire genome, the technology could theoretically provide PGT for all patients with single-gene diseases that have clear pathogenic genes and complete pedigree information.

For PGT-M, the advantages of using a karyomap chip are apparent. An informational SNP marker set related to the pathogenic gene was determined through the detection of SNP polymorphic sites in the foetus, the parents carrying the pathogenic gene and a parental progenitor, and the haplotype of each embryo was constructed from the detected SNP locus information via the linkage analysis method to determine whether the pathogenic gene fragment in the family was inherited. This technique can minimize the ADO caused by whole genome amplification [15, 28]. In addition, karyomapping can directly select pathogenic genes or adjacent regions for SNP analysis, without the need to design the customized STR sites; thus, the detection process is more convenient. Multiple types of variation information can be obtained at the same time to detect chromosome aneuploidy, copy number variation (CNV) [29, 30]. Therefore, karyomapping is an ideal and feasible strategy.

## Conclusions

In summary, we reported an effective and feasible PGT-M strategy based on karyomapping linkage analysis to identify the carrier status of pathogenic gene mutations in embryos; three healthy infants were born, demonstrating the effectiveness of this method. In this study, five variants in three pathogenic genes (IL2RG, RAG2, and LIG4) in four SCID families were detected, three of which were novel variants. We confirmed that the PGT-M strategy, which is based on linkage analysis by karyomapping, is an efficient and valuable method for PGT-M. This technology can effectively block the vertical transmission of familial genetic diseases.

## Electronic supplementary material

Below is the link to the electronic supplementary material.

Supplementary Material 1

Supplementary Material 2

Supplementary Material 3

Supplementary Material 4

Supplementary Material 5

Supplementary Material 6

## Data Availability

The data that support the results of this study can be obtained from the corresponding author, upon reasonable request.

## Abbreviations

SCID: Severe combined immunodeficiency
PGT: Preimplantation genetic testing
PGT-M: Preimplantation genetic testing for monogenic defects
MDA: Multiple displacement amplification
SNP: Single nucleotide polymorphism
ART: Assisted reproductive technology
FET: Frozen embryo transfer
ACMG: The American College of Medical Genetics and Genomics
WGA: Whole genome amplification
TE: Trophoblastic ectodermal
CNV: Copy number variation
ICSI: Intracytoplasmic sperm injection
HSCT: Hematopoietic stem cell transplantation
IL2RG: Interleukin 2 receptor gamma
JAK3: Janus family tyrosine kinase 3
IL-7Rα: Interleukin 7 receptor alpha chain
RAG1: Recombination activating gene 1
LIG4: DNA ligase IV
WES: Whole exome sequencing
NGS: Next-generation sequencing
REVEL: Rare Exome Variant Ensemble Learner
ADO: Allele drop out
