# Supplementary material for: Preimplantation genetic testing for four families with severe combined immunodeficiency: Three unaffected livebirths
Source: Orphanet J Rare Dis. 2025 Jan 9;20:14. doi: 10.1186/s13023-024-03525-y (PMC11720562; doi:10.1186/s13023-024-03525-y)
Supplement: Supplementary file 1 — Supplementary Material 1 [file 13023_2024_3525_MOESM1_ESM.docx]

**Table S1 Pathogenic gene information included in SCID cases**

| **Case ID** | **Gene** | **Phenotype MIM number** | **Location** | **Start Position** | **Stop Position** | **Length** | **Analysis zone** |
| --- | --- | --- | --- | --- | --- | --- | --- |
| 1 | IL2RG | 300400 | Xq13.1 | 71107404 | 71111577 | 4.2kb | 70107404-72111577 |
| 2 | IL2RG | 300400 | Xq13.1 | 71107404 | 71111577 | 4.2kb | 70107404-72111577 |
| 3 | RAG2 | 602450 | 11p12 | 36590996 | 36598236 | 7.2kb | 35590996-37598326 |
| 4 | LIG4 | 606593 | 13q33.3 | 108207442 | 108218349 | 10.9kb | 107207442-109218349 |

Abbreviation: SCID, Severe combined immunodeficiency, MIM, Mendelian Inheritance in Man.
