# Supplementary material for: Preimplantation genetic testing for four families with severe combined immunodeficiency: Three unaffected livebirths
Source: Orphanet J Rare Dis. 2025 Jan 9;20:14. doi: 10.1186/s13023-024-03525-y (PMC11720562; doi:10.1186/s13023-024-03525-y)
Supplement: Supplementary file 2 — Supplementary Material 2 [file 13023_2024_3525_MOESM2_ESM.docx]

**Table S2 Informative SNPs flanking IL2RG gene of SCID in Case1（Reference：Couples' son）**

| **Probe ID** | **Chr** | **Position** | **Informative** | **Male** | **Female** | **Reference** | **E1** | **E2** | **E3** | **E4** | **E5** | **E6** | **E7** | **E8** |
| --- | --- | --- | --- | --- | --- | --- | --- | --- | --- | --- | --- | --- | --- | --- |
| rs2369216 | X | 71878640 | Mother informative | BB | AB | AA | AA | AA | AB | BB | AB | AB | AA | BB |
| rs2272781 | X | 71876171 | Mother informative | BB | AB | AA | AA | AA | AB | BB | AB | AB | AA | BB |
| rs3788796 | X | 71870538 | Mother informative | AA | BA | BB | BB | BB | BA | AA | BA | BA | BB | AA |
| rs4585883 | X | 71867586 | Mother informative | BB | AB | AA | AA | AA | AB | BB | AB | / | AA | BB |
| rs576502 | X | 71561373 | Mother informative | AA | AB | AA | AA | AA | AA | AB | AA | AA | AA | AB |
| rs6525505 | X | 71034614 | Mother informative | AA | AB | AA | AA | AA | AA | AB | AA | / | AA | AB |
| rs12387850 | X | 71024480 | Mother informative | BB | BA | BB | BB | BB | BB | AB | BB | BB | BB | AB |
| rs5937104 | X | 70872967 | Mother informative | BB | BA | BB | BB | BB | BB | AB | BB | BB | BB | AB |
| rs6625811 | X | 70855518 | Mother informative | AA | AB | AA | AA | AA | AA | AB | AA | AA | AA | AB |
| rs35384418 | X | 70782669 | Mother informative | AA | AB | AA | AA | AA | AA | AB | AA | AA | AA | AB |
| rs4844285 | X | 70370244 | Mother informative | BB | BA | BB | BB | BB | BB | AB | BB | BB | BB | AB |
| rs5981066 | X | 70294222 | Mother informative | AA | AB | AA | AA | AA | AA | AB | AA | AA | AA | AB |
| rs3125945 | X | 70258736 | Mother informative | AA | AB | AA | AA | AA | AA | AB | AA | AA | AA | AB |
| rs5937054 | X | 70220983 | Mother informative | BB | BA | BB | BB | BB | BB | AB | BB | BB | BB | AB |
| SNP, single nucleotide polymorphism; Chr, chromosome; E, embryo; “/” not available.  Red font indicates SNPs associated with pathogenic mutation | | | | | | | | | | | | | | |
