# Supplementary material for: Preimplantation genetic testing for four families with severe combined immunodeficiency: Three unaffected livebirths
Source: Orphanet J Rare Dis. 2025 Jan 9;20:14. doi: 10.1186/s13023-024-03525-y (PMC11720562; doi:10.1186/s13023-024-03525-y)
Supplement: Supplementary file 3 — Supplementary Material 3 [file 13023_2024_3525_MOESM3_ESM.docx]

**Table S3 Informative SNPs flanking IL2RG gene of SCID in Case2（Reference：Female's mother）**

| **Probe ID** | **Chr** | **Position** | **Informative** | **Male** | **Female** | **Reference** | **E1** | **E2** | **E3** | **E4** | **E5** |
| --- | --- | --- | --- | --- | --- | --- | --- | --- | --- | --- | --- |
| rs4844279 | X | 70142581 | Mother informative | BB | AB | AA | / | AA | BB | BB | BB |
| rs5937039 | X | 70184830 | Mother informative | AA | BA | BB | BA | BB | AA | AA | AA |
| rs5937054 | X | 70220983 | Mother informative | BB | AB | AA | AB | AA | BB | BB | BB |
| rs3125945 | X | 70258736 | Mother informative | AA | BA | BB | BA | BB | AA | AA | AA |
| rs4844285 | X | 70370244 | Mother informative | AA | AB | AA | AA | AA | BB | AB | BB |
| rs4844146 | X | 70410500 | Mother informative | AA | AB | AA | AA | AA | BB | AB | BB |
| rs12387850 | X | 71024480 | Mother informative | BB | BA | BB | BB | BB | AA | BA | AA |
| rs6525505 | X | 71034614 | Mother informative | AA | AB | AA | AA | AA | BB | / | BB |
| There are 8 SNPs informative SNPs for linkage analyses | | | |  |  |  |  |  |  |  |  |
| SNP, single nucleotide polymorphism; Chr, chromosome; E, embryo; “/” not available. | | | | | | | | | | | |
| Red font indicates SNPs associated with pathogenic mutation | | | |  |  |  |  |  |  |  |  |
