# Supplementary material for: Preimplantation genetic testing for four families with severe combined immunodeficiency: Three unaffected livebirths
Source: Orphanet J Rare Dis. 2025 Jan 9;20:14. doi: 10.1186/s13023-024-03525-y (PMC11720562; doi:10.1186/s13023-024-03525-y)
Supplement: Supplementary file 4 — Supplementary Material 4 [file 13023_2024_3525_MOESM4_ESM.docx]

**Table S4 Informative SNPs flanking RAG2 gene of SCID in Case3（Reference：Male's mother）**

| **Probe ID** | **Chr** | **Position** | **Informative** | **Male** | **Female** | **Reference** | **E1** | **E2** | **E3** | **E4** | **E5** |
| --- | --- | --- | --- | --- | --- | --- | --- | --- | --- | --- | --- |
| rs4756394 | 11 | 37344446 | Father informative | AB | AA | BB | BA | BA | BA | AA | BA |
| rs1949703 | 11 | 37291017 | Father informative | AB | BB | BB | BB | BB | BB | / | BB |
| rs11034056 | 11 | 37285494 | Father informative | AB | AA | AA | AA | AA | AA | / | AA |
| rs6484915 | 11 | 37283532 | Father informative | AB | AA | AA | AA | AA | AA | / | AA |
| rs11034037 | 11 | 37256343 | Father informative | AB | AA | AA | AA | AA | AA | AB | AA |
| rs4756376 | 11 | 37249967 | Father informative | AB | BB | AA | AB | AB | AB | BB | AB |
| rs1825015 | 11 | 36960292 | Father informative | AB | AA | AA | AA | AA | AA | AB | AA |
| rs10836640 | 11 | 36952592 | Father informative | AB | AA | AA | AA | AA | AA | / | AA |
| rs1383349 | 11 | 36938773 | Father informative | AB | AA | AA | AA | AA | AA | AB | AA |
| rs4756321 | 11 | 36466141 | Father informative | AB | BB | AA | AB | AB | AB | BB | / |
| rs10742373 | 11 | 36465453 | Father informative | AB | AA | BB | BA | BA | BA | AA | / |
| rs1863398 | 11 | 36453978 | Father informative | AB | AA | BB | BA | BA | BA | AA | BA |
| rs744895 | 11 | 36448379 | Father informative | AB | AA | AA | AA | AA | AA | AB | AA |
| rs330260 | 11 | 36422172 | Father informative | AB | BB | AA | AB | AB | AB | BB | AB |
| rs3812766 | 11 | 36294052 | Father informative | AB | AA | AA | AA | AA | AA | AB | AA |
| rs2289989 | 11 | 36290278 | Father informative | AB | BB | AA | AB | AB | AB | BB | AB |
| rs12576603 | 11 | 36178090 | Father informative | AB | BB | BB | BB | BB | BB | AB | BB |
| rs2422179 | 11 | 36175645 | Father informative | AB | BB | BB | BB | BB | BB | AB | BB |
| rs2422178 | 11 | 36155578 | Father informative | AB | BB | AA | AB | AB | AB | BB | AB |
| rs11033448 | 11 | 36154375 | Father informative | AB | AA | BB | BA | BA | BA | AA | BA |
| rs12226262 | 11 | 35875576 | Father informative | AB | BB | BB | BB | BB | BB | / | BB |
| SNP, single nucleotide polymorphism; Chr, chromosome; E, embryo; “/” not available.  Red font indicates SNPs associated with pathogenic mutation | | | | | | | | | | | |
