# Supplementary material for: Preimplantation genetic testing for four families with severe combined immunodeficiency: Three unaffected livebirths
Source: Orphanet J Rare Dis. 2025 Jan 9;20:14. doi: 10.1186/s13023-024-03525-y (PMC11720562; doi:10.1186/s13023-024-03525-y)
Supplement: Supplementary file 5 — Supplementary Material 5 [file 13023_2024_3525_MOESM5_ESM.docx]

**Table S5 Informative SNPs flanking RAG2 gene of SCID in Case3（Reference：Female's mother）**

| **Probe ID** | **Chr** | **Position** | **Informative** | **Male** | **Female** | **Reference** | **E1** | **E2** | **E3** | **E4** | **E5** |
| --- | --- | --- | --- | --- | --- | --- | --- | --- | --- | --- | --- |
| rs1461898 | 11 | 37590232 | Mother informative | BB | AB | AA | AB | BB | AB | AB | BB |
| rs3980290 | 11 | 37577216 | Mother informative | BB | AB | AA | AB | BB | AB | AB | BB |
| rs7931655 | 11 | 37573427 | Mother informative | AA | BA | BB | BA | AA | BA | BA | AA |
| rs10836754 | 11 | 37569819 | Mother informative | BB | AB | AA | AB | BB | AB | AB | BB |
| rs4630265 | 11 | 37521486 | Mother informative | AA | BA | BB | BA | / | BA | / | AA |
| rs11606085 | 11 | 37489822 | Mother informative | AA | BA | BB | BA | AA | BA | BA | AA |
| rs987827 | 11 | 37426627 | Mother informative | BB | AB | AA | AB | / | AB | AB | BB |
| rs10836736 | 11 | 37406069 | Mother informative | AA | BA | BB | BA | AA | / | BA | AA |
| rs1522679 | 11 | 37350828 | Mother informative | AA | BA | BB | BA | / | BA | BA | AA |
| rs9633876 | 11 | 37252050 | Mother informative | AA | AB | AA | AA | AB | AA | AA | AB |
| rs1512368 | 11 | 37115843 | Mother informative | AA | AB | AA | AA | AB | AA | AA | AB |
| rs10768243 | 11 | 37090499 | Mother informative | AA | AB | AA | AA | AB | AA | AA | / |
| rs2089917 | 11 | 37063291 | Mother informative | BB | BA | BB | BB | AB | BB | BB | AB |
| rs333810 | 11 | 37045724 | Mother informative | AA | AB | AA | AA | AB | / | AA | AB |
| rs333805 | 11 | 37043351 | Mother informative | AA | AB | AA | AA | AB | / | AA | AB |
| rs333822 | 11 | 37020611 | Mother informative | AA | AB | AA | AA | AB | AA | AA | AB |
| rs11033786 | 11 | 36766061 | Mother informative | BB | BA | BB | BB | AB | BB | BB | AB |
| rs964527 | 11 | 36764288 | Mother informative | AA | AB | AA | AA | / | AA | AA | AB |
| rs1914762 | 11 | 36747878 | Mother informative | BB | BA | BB | BB | AB | BB | BB | AB |
| rs7929580 | 11 | 36458617 | Mother informative | AA | AB | AA | AA | AB | / | AA | AB |
| rs10501155 | 11 | 36445197 | Mother informative | BB | BA | BB | BB | AB | / | BB | AB |
| rs10836550 | 11 | 36427893 | Mother informative | AA | BA | BB | BA | AA | / | BA | AA |
| rs11033564 | 11 | 36367117 | Mother informative | BB | BA | BB | BB | / | BB | BB | AB |
| rs11033559 | 11 | 36356259 | Mother informative | AA | AB | AA | AA | AB | / | AA | AB |
| rs11033553 | 11 | 36349396 | Mother informative | BB | BA | BB | BB | AB | / | BB | AB |
| rs7109505 | 11 | 36288752 | Mother informative | BB | BA | BB | BB | AB | BB | BB | AB |
| rs11551023 | 11 | 36251811 | Mother informative | AA | BA | BB | BA | AA | BA | / | AA |
| rs11605931 | 11 | 36237763 | Mother informative | BB | AB | AA | AB | BB | AB | AB | BB |
| rs3812772 | 11 | 36218218 | Mother informative | AA | BA | BB | BA | AA | BA | BA | AA |
| rs4755440 | 11 | 36202307 | Mother informative | AA | BA | BB | BA | AA | BA | BA | AA |
| rs882366 | 11 | 36062969 | Mother informative | BB | AB | AA | AB | BB | AB | AB | BB |
| rs7947746 | 11 | 36033069 | Mother informative | BB | AB | AA | AB | BB | AB | AB | BB |
| rs12274406 | 11 | 36020797 | Mother informative | AA | BA | BB | BA | AA | BA | BA | AA |
| rs16928055 | 11 | 35913571 | Mother informative | AA | AB | AA | AA | AB | AA | AA | AB |
| rs7129170 | 11 | 35754464 | Mother informative | AA | AB | AA | AA | AB | AA | AA | AB |
| rs16927798 | 11 | 35657763 | Mother informative | AA | AB | AA | AA | AB | AA | AA | AB |
| rs579687 | 11 | 35629494 | Mother informative | BB | BA | BB | BB | AB | / | BB | AB |
| SNP, single nucleotide polymorphism; Chr, chromosome; E, embryo; “/” not available.  Red font indicates SNPs associated with pathogenic mutation | | | | | | | | | | | |
