# Supplementary material for: Preimplantation genetic testing for four families with severe combined immunodeficiency: Three unaffected livebirths
Source: Orphanet J Rare Dis. 2025 Jan 9;20:14. doi: 10.1186/s13023-024-03525-y (PMC11720562; doi:10.1186/s13023-024-03525-y)
Supplement: Supplementary file 6 — Supplementary Material 6 [file 13023_2024_3525_MOESM6_ESM.docx]

**Table S6 Informative SNPs flanking LIG4 gene of SCID in Case4（Reference：Couples' son）**

| **Probe ID** | **Chr** | **Position** | **Informative** | **Male** | **Female** | **Reference** | **E1** | **E2** | **E3** | **E4** | **E5** |
| --- | --- | --- | --- | --- | --- | --- | --- | --- | --- | --- | --- |
| rs4772979 | 13 | 109221219 | Mother informative | AA | AB | AA | AB | AA | AA | BB | AB |
| rs7321084 | 13 | 109173965 | Mother informative | BB | BA | BB | / | BB | BB | AA | / |
| rs1951896 | 13 | 109116454 | Mother informative | BB | BA | BB | / | BB | / | / | / |
| rs9587585 | 13 | 109090867 | Mother informative | AA | AB | AA | AB | AA | AA | BB | AB |
| rs9301295 | 13 | 109066918 | Mother informative | AA | AB | AA | AB | AA | AA | BB | AB |
| rs16972387 | 13 | 109060141 | Mother informative | BB | BA | BB | / | BB | BB | AA | AB |
| rs9559323 | 13 | 109046867 | Mother informative | BB | BA | BB | AB | BB | BB | AA | AB |
| rs17381815 | 13 | 109015760 | Mother informative | AA | AB | AA | AB | AA | AA | BB | AB |
| rs1151403 | 13 | 108858373 | Mother informative | BB | BA | BB | AB | BB | BB | / | / |
| rs11620361 | 13 | 108857648 | Mother informative | BB | BA | BB | AB | BB | BB | AA | / |
| rs10492664 | 13 | 108816225 | Mother informative | BB | BA | BB | AB | BB | BB | AA | AB |
| rs1325385 | 13 | 108612706 | Mother informative | AA | AB | AA | AB | AA | AA | BB | / |
| rs2136267 | 13 | 108534782 | Mother informative | BB | BA | BB | AB | / | BB | AA | / |
| rs9559160 | 13 | 108448355 | Mother informative | AA | AB | AA | AB | AA | AA | BB | / |
| rs1547801 | 13 | 108416500 | Mother informative | AA | AB | AA | AB | AA | AA | / | AB |
| rs2146690 | 13 | 108403984 | Mother informative | AA | AB | AA | AB | AA | AA | / | AB |
| rs9520552 | 13 | 108339060 | Mother informative | AA | AB | AA | AB | AA | AA | BB | AB |
| rs9520551 | 13 | 108338976 | Mother informative | AA | AB | AA | AB | AA | AA | BB | AB |
| rs4083987 | 13 | 108332559 | Mother informative | AA | AB | AA | AB | AA | AA | / | AB |
| rs9587389 | 13 | 108082350 | Mother informative | BB | BA | BB | AB | BB | BB | AA | / |
| rs16970077 | 13 | 108064103 | Mother informative | BB | BA | BB | AB | BB | BB | AA | / |
| rs9583173 | 13 | 108064056 | Mother informative | AA | AB | AA | AB | / | AA | BB | AB |
| rs12583955 | 13 | 107983134 | Mother informative | AA | AB | AA | AB | AA | AA | BB | AB |
| rs9583163 | 13 | 107939161 | Mother informative | AA | AB | AA | AB | AA | AA | BB | AB |
| rs10508184 | 13 | 107840018 | Mother informative | AA | AB | AA | AB | AA | AA | BB | AB |
| rs2150171 | 13 | 107552969 | Mother informative | BB | BA | BB | AB | BB | BB | AA | AB |
| rs16968967 | 13 | 107300200 | Mother informative | AA | AB | AA | AB | AA | AA | BB | AB |
| rs16968951 | 13 | 107296805 | Mother informative | BB | BA | BB | AB | BB | BB | AA | AB |
| rs9558802 | 13 | 107277967 | Mother informative | AA | AB | AA | AB | AA | / | BB | / |
| rs11069652 | 13 | 107257959 | Mother informative | AA | AB | AA | AB | AA | AA | BB | / |
| SNP, single nucleotide polymorphism; Chr, chromosome; E, embryo; “/” not available.  Red font indicates SNPs associated with pathogenic mutation | | | | | | | | | | | |
